# Generative adversarial networks for balancing and expanding data resources for computer-aided detection in colonoscopy

**DOI:** 10.3389/fmedt.2026.1824811

**Published:** 2026-07-09

**Authors:** J. J. H. van der Laan, J. van Lune, L. R. B. Schomaker, P. M. A. van Ooijen, W. B. Nagengast

**Affiliations:** 1 Department of Gastroenterology and Hepatology, University Medical Center Groningen, University of Groningen, Groningen, Netherlands; 2 Department of Radiation Oncology, University Medical Center Groningen, University of Groningen, Groningen, Netherlands; 3 Faculty of Science and Engineering, University of Groningen, Groningen, Netherlands; 4 Computational Imaging Group for MRI Therapy & Diagnostics, University Medical Center Utrecht, Utrecht, Netherlands

**Keywords:** adenomas, colonoscopy, computer-aided detection, generative AI, synthetic data

## Abstract

**Background and aims:**

Developing computer-aided detection (CADe) algorithms in colonoscopy requires high-quality databases sourced from real-world data. However, such databases demand costly investments and display imbalanced distributions that underrepresent flat adenomas. Therefore, we evaluated whether StyleGAN2-ADA, a generative adversarial network, can generate synthetic images as alternative training resources for CADe, and whether a modified StyleGAN2-ADA can enhance synthesis control for better balanced datasets.

**Methods:**

Two synthetic datasets were generated using the original StyleGAN2-ADA and our modified version with feature-clustered conditioning vectors. Generative adversarial networks were trained on images from our local colonoscopy database, and their outputs were evaluated through Fréchet Inception Distance where lower scores indicate more realistic and diverse data. Three CADe models were trained using synthetic or real-world data and tested in two external databases comprising only polyp images. CADe performance included mean average precision (mAP) score as critical metric for polyp identification.

**Results:**

The modified StyleGAN2-ADA achieved a lower Fréchet Inception Distance-score (7.54 vs. 13.68) than the original StyleGAN2-ADA. During external testing, CADe trained on synthetic data from the modified version achieved performance comparable to the model trained on real-world data across all metrics (*p* > 0.05). It also outperformed the model trained on synthetic data from the original StyleGAN2-ADA, achieving mAP-scores of 0.77 ± 0.03 vs. 0.64 ± 0.02 in the first test set (*p* < 0.001) and 0.91 ± 0.02 vs. 0.87 ± 0.02 in the second (*p* = 0.012).

**Conclusions:**

StyleGAN2-ADA incorporating feature-clustered conditioning vectors can synthesize better balanced colonoscopy databases that offer alternative training resources to real-world data for CADe training and validation.

## Introduction

Colonoscopy is the foremost area in gastroenterology for the integration of artificial intelligence. Numerous studies have investigated the use of deep learning (DL) networks for computer-aided detection (CADe) of colorectal cancer (CRC) and its premalignant adenomatous polyps. Overall, CADe results in an increase in the adenoma detection rate (ADR), a metric associated with the risk of developing post-colonoscopy CRC (1). However, current algorithms persistently fail to detect flat adenomas, particularly the advanced ones, which concern clinically significant lesions that should be identified by CADe as they are prone to be missed by the human eye due to their subtle morphology (2).

High-quality databases containing diverse endoscopic images are essential for the training and validation of CADe algorithms. However, constructing such databases is both labor-intensive and time-consuming as the images are typically collected from routine colonoscopies and require manual annotation by expert endoscopists (3). Publicly available databases have therefore been introduced, but these frequently provide incomplete metadata, which reduces their suitability for algorithm development (4). While detailed metadata can enhance dataset utility, it also raises the risk of patient traceability. This may create conflicts with privacy legislation, thereby limiting data sharing for algorithm training and validation (5). Another limitation of clinically sourced databases is the imbalanced data distribution of polyp morphologies, reflecting their prevalence in clinical practice. As a result, flat adenomas are typically underrepresented, including in publicly available databases, thereby limiting the ability of CADe systems to consistently detect these lesions (6).

To expand data resources for training and validating CADe algorithms, the creation of synthetic images by generative adversarial networks (GANs) has been broadly proposed across several medical imaging domains (7, 8). A GAN comprises two neural networks, a generator and a discriminator, that are trained simultaneously. The generator synthesizes images from randomly sampled vectors within a latent space: a mathematical domain where each point represents a potential new image. The discriminator trains to distinguish synthetic images from real-world images drawn from a reference database and also provides feedback that helps the generator improve. During early training stages, the generator produces low-quality images that the discriminator can usually differentiate from real ones. As training progresses, the generator gradually refines its outputs, ultimately synthesizing images that can increasingly fool the discriminator (9). Consequently, GANs can generate large volumes of high-quality data for training and validating CADe algorithms that are not directly tied to real individuals, thereby helping to mitigate barriers to data sharing. Yet, the potential of synthetic datasets as readily shareable surrogates for current databases that maintain CADe performance has not been evaluated. Beyond facilitating data sharing, GANs could be used to correct imbalanced distribution within training datasets by synthesizing targeted samples of underrepresented cases to resolve detection blind spots for CADe algorithms. However, conventional generative architectures lack explicit mechanisms to control the characteristics of generated outputs during synthesis. As a consequence, standard GANs tend to reproduce the class distributions of their source databases rather than purposefully enriching them with underrepresented cases. Thus, they perpetuate the very data imbalances they were intended to address (10).

Therefore, this study evaluated the feasibility of two GAN models to generate synthetic endoscopy images of colorectal polyps as high-quality alternatives to databases sourced from real-world images for CADe training. Specifically, we compared an original StyleGAN2-ADA with a modified version utilizing feature-clustered conditioning vectors designed to purposefully control polyp morphology and address class imbalance. To investigate these strategies, the performance of CADe models under three training conditions, one real-world dataset and two synthetically generated datasets by the original and modified StyleGAN2-ADA, were tested in two external databases. The synthetic datasets were also evaluated on their balance regarding polyp morphology, with particular attention to the representation of flat polyps. Additionally, we exploratively examined whether augmenting training datasets with synthetic images of hard-to-detect polyps could enhance CADe performance.

## Methods

### Study design

We conducted a retrospective study on images from colonoscopies with polypectomies in patients that were admitted to the gastroenterology department of the University Medical Center Groningen (UMCG). The research protocol for using colonoscopy data was approved by the UMCG institutional review board (202100575). Informed consent of patients was waived due to the retrospective nature and the sample size of the study. Before data inclusion, we checked the local UMCG registry for objections by patients regarding usage of their data for research purposes.

### Synthetic data generation

We selected StyleGAN2-ADA (11) as GAN for synthetic colonoscopy image generation due to its state-of-the-art performance and its effectiveness with limited training data. To provide appropriate training data, we constructed a local UMCG database by reviewing 10,525 images from 4,222 colonoscopies with registered polypectomies performed in 2,982 anonymized patients from 2011 to 2021. After excluding images with chromoendoscopy, artifacts, or endoscopic instruments, 3,681 white-light endoscopy images remained, of which 2,189 contained polyps (Table 1). All images were acquired using endoscopes from the Olympus 180 and 190-CF series. As detailed in Table 1, the baseline distribution of these polyps was imbalanced, favoring sessile polyps (*N* = 2071) over flat (*N* = 99) and pedunculated (*N* = 19) morphologies**.** To mitigate this class imbalance prior to GAN training, the UMCG database underwent a non-destructive augmentation approach. While augmentation was applied across all classes, the underrepresented flat and pedunculated polyps received significantly higher augmentation factors. For each of the 99 authentic flat images, 10 new images were generated, which resulted in a total of 1,089 images. Similarly, for each of the 19 pedunculated images, 20 new images were created, yielding a total of 399 images. This approach allowed the GAN to access to a final set of 8,614 images to learn minority class features (Supplementary Material S1).

**Table 1:** Specifications of the three databases containing real-world images.

**Database**	**Images**	**Patients**	**Colonoscopies**	**Unique polyps**	**Polyp distribution** ^a^	
UMCG	3,681	2,982	4,222	Unknown^b^	No polyp	1,492
					Flat	99
					Sessile	2m071
					Pedunculated	19
SUN	49,136	99	Unknown	100	Protruding	66
					Flat	34
PIBA	1,030	unknown	59	95	Sessile	53
					Slightly elevated	12
					Pedunculated	30

a Morphology distributions were derived from database-specific annotation systems; therefore, they are not similar across all datasets.

b Labels follow the original database annotations; counts of unique polyps per label are not specified.

**Figure 1:**
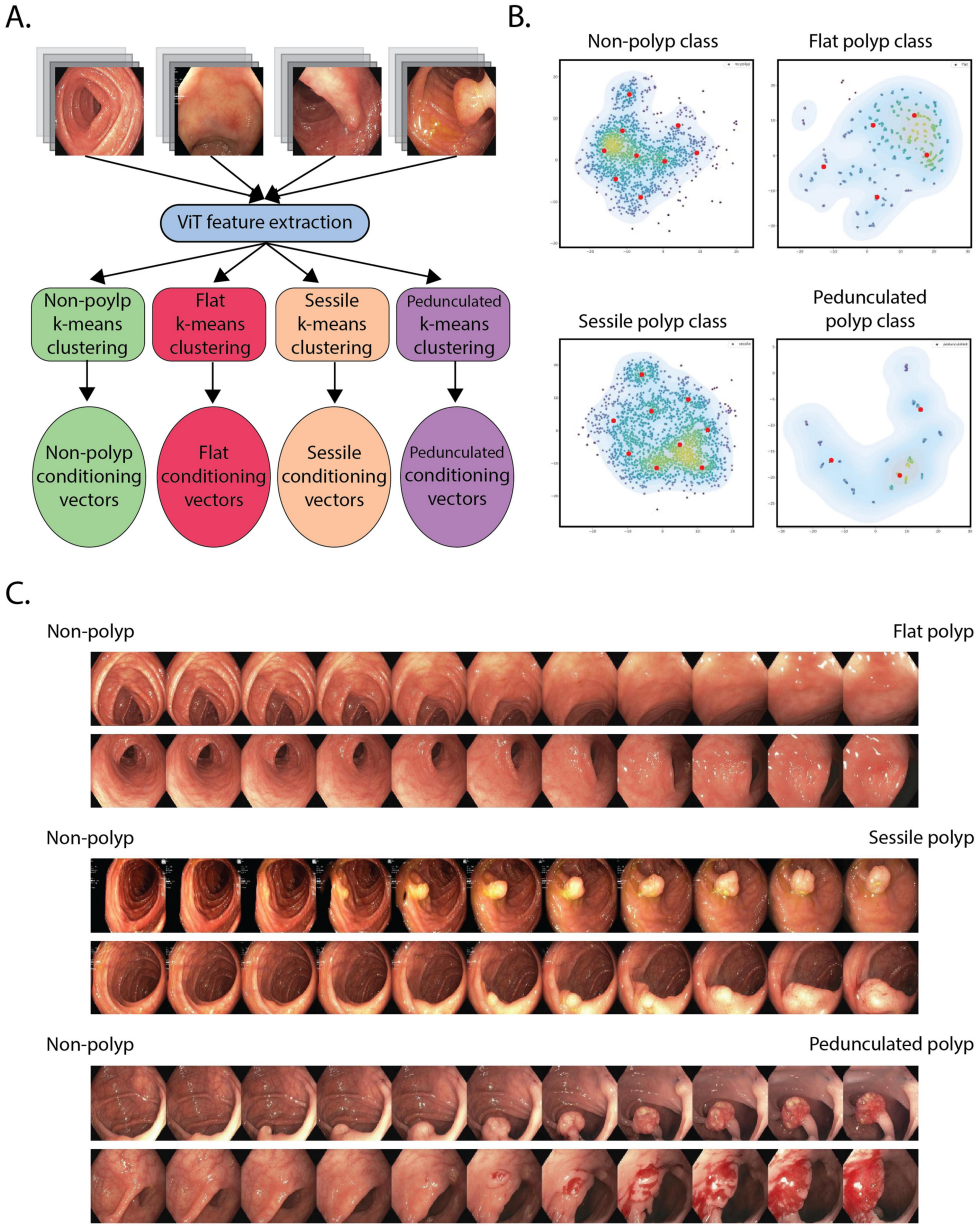
Overview of the design of the conditional GAN. **(A)** Workflow to establish the conditioning vectors based on k-means clustering of extracted ViT features from the colonoscopy images. **(B)** Two-dimensional spaces of the distribution of image features were created using t-distributed stochastic neighbor embedding (t-SNE) visualizations in kernel density (KDE) plots for each polyp morphology. The k-means centroids found in this space are plotted as red dots and were used to determine the appropriate number of image feature clusters per morphology class. **(C)** Interpolation results of images generated directly from a non-polyp centroid to sessile/pedunculated/flat centroids. Every row contains a linear interpolation in the W-space (post-mapping network in StyleGAN2-ADA). The images in between are generated with mixing parameter steps of 0.10.

To gain increased control over the generation process and construct a more diverse dataset regarding polyp morphology, we modified StyleGAN2-ADA by incorporating feature-clustered conditioning vectors. In this approach, characteristic visual features were first identified and extracted from all images of the UMCG database using a vision transformer (ViT) model (12) (Supplementary Material S2). These image features were then clustered within each polyp class (non-polyp, flat, sessile, and pedunculated) using *k*-means clustering to create separate subclasses that capture specific variations in polyp morphology (Figure 1A). To determine the optimal number of clusters per class, we assessed the distribution of the image features across a plotted two-dimensional space (Figure 1B) (13). Based on the coverage of the centroids, which represent the average position of a cluster in image feature space, we selected nine feature clusters for both the non-polyp and sessile classes, five for the flat class, and three for the pedunculated class. Feature clusters from the flat, sessile, and pedunculated polyp classes served as conditioning vectors for generating synthetic images within their respective classes, whereas feature clusters from the non-polyp class were only used for linear interpolation in the post-mapping network latent space to enhance image diversity (Figure 1C). Additionally, we aimed to optimize generation outcomes by the modified StyleGAN2-ADA through three additional technical adjustments: replacing logistic loss with hinge loss (14), introducing a classifier guidance loss term, and adopting the *Stylesplit* method (15) for more effective conditional embedding fusion (Supplementary Material S3). Eventually, we generated two synthetic datasets for CADe training: one generated by the original StyleGAN2-ADA, synthesizing images from random latent vectors, and one generated by the modified StyleGAN2-ADA, synthesizing images by conditioning the generation process on the feature-clustered conditioning vectors. Both GANs were trained using the UMCG database and their training details are described in Supplementary Material S4.

The synthetic images generated by both GANs were jointly reviewed by two medical experts to select those judged to be high quality and realistic. The selected images were then manually annotated with bounding boxes around the polyps. Additionally, the polyp morphology of the synthetic image was documented by the same reviewers according to the Paris endoscopic polyp classification (16). We aimed to create synthetic datasets containing approximately 80% of the images in the UMCG database, corresponding to the training set size used for CADe in five-fold cross-validation.

### Training and validating CADe

YOLOv5m (17) was employed as CADe algorithm for identifying and locating the polyps in the colonoscopy images due to its previously reported high performance in colonoscopy polyp detection (18, 19). The model was pretrained on the COCO database (20) and then trained under three different conditions: real-world data from the UMCG database, synthetic data from the original StyleGAN2-ADA, and synthetic data from the modified StyleGAN2-ADA. The performances of these training strategies were evaluated in three separate databases: internally in the UMCG database and externally in the publicly available SUN (21, 22) and PIBA (23) databases (Table 1). The SUN and PIBA databases comprised exclusively images with polyps. All images in the databases were labeled with bounding box coordinates to localize the polyp in the image. Similar to the UMCG database, images with instruments or in-screen snapshots were removed from the PIBA database; this was not done in the SUN database due to the high number of images.

**Figure 2:**
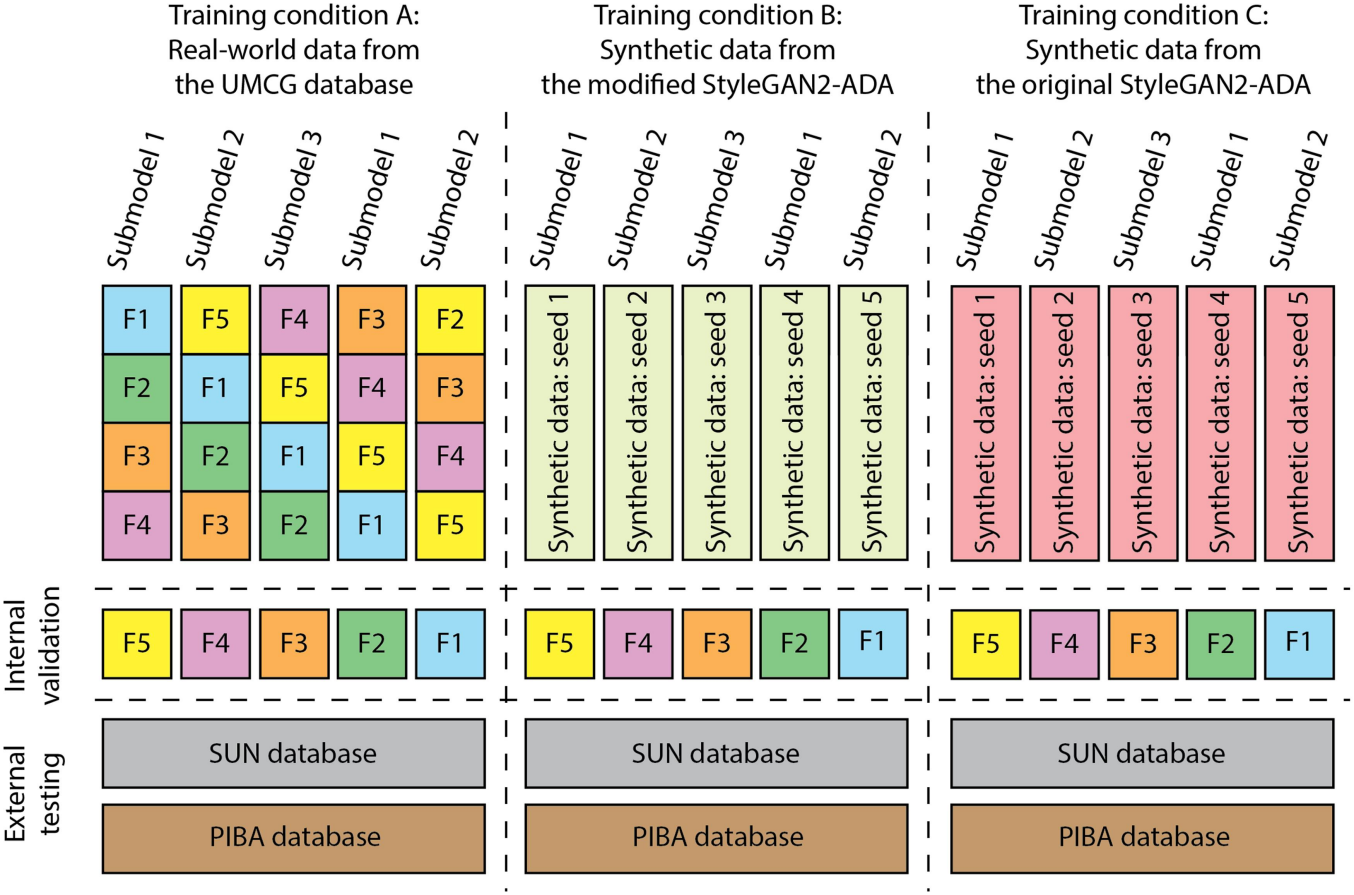
Schematic overview of training the five submodels for each of the three training conditions for internal validation and external testing. **(A)** The model trained on the real-world data was internally validated using five-fold cross-validation. **(B)** The same validation folds were applied to assess the performances of the YOLOv5m models that were trained on synthetic data. **(C)** The real-world training folds were augmented with synthetic images prior to internal validation to assess the yield of synthetic data augmentation. All models were externally tested in the SUN and PIBA databases.

To investigate the primary aim, we compared the performances of YOLOv5m under the three training conditions during external testing after internal validation. Thus, we trained the model on real-world data from the UMCG database using five-fold cross-validation: the database of 2,189 images containing polyps was randomly split into five folds (four folds contained 438 images and one fold contained 437 images). Because mandatory anonymization precluded patient-level separation, we utilized sample-level splitting. To account for the resulting risk of internal cross-fold data leakage, performance on the external databases served as our definitive evaluation metric. Each fold served once as the internal validation set for a submodel trained on the four remaining folds (Figure 2A). These validation folds were also used to evaluate the performances of the YOLOv5m models that were trained on synthetic data (Figure 2B). Training of these models was performed in five separate runs, each using all synthetic images with different random seeds set at training time. Subsequently, the three YOLOv5m models were tested externally in the SUN database (49,136 images containing 100 unique polyps) and PIBA database (1,030 images containing 95 unique polyps), utilizing the same five submodels from each condition as those evaluated during internal validation.

To exploratively assess the yield of synthetic data augmentation, we used the same fold partitions of the UMCG database as in the primary analysis and augmented the four training folds for each submodel with synthetic images. Each submodel was internally validated on the corresponding held-out UMCG fold and tested externally on the PIBA and SUN databases to assess the effect of synthetic data augmentation on the detection performance (Figure 2C). In the external databases, we visually assessed (“eyeballed”) which polyp classes were difficult to detect for the YOLOv5 model trained on real-world data and considered these as hard-to-detect lesions. We then augmented the training set with synthetic images corresponding to these hard-to-detect polyp classes. In all training experiments, we applied traditional data augmentation on all data, including the synthetically generated (see for specifications Supplementary Material S5).

### Outcome measures and statistics

We report the Fréchet Inception Distance (FID) score to assess the quality of synthetic images created by GANs (24). The FID score compares the distribution of image features extracted from synthetic images with those from real images in the reference database, using a pre-trained neural network. Lower FID scores indicate good image quality and sufficient diversity, suggesting that the synthetic images are more similar to real images.

Predictions made by YOLOv5m were defined as true positives when the predicted bounding box around a polyp overlapped with at least 50% of the area of the reference bounding box drawn by a medical expert (Intersection over Union ≥ 0.5). If this overlap was less than 50%, the prediction was considered a false positive (FP). Images in which YOLOv5m did not detect a polyp were counted as false negatives (FN). The detection models were not evaluated on images without polyps as these were not present in the external databases. Detection performances are assessed using the positive predictive value (PPV), the sensitivity, their harmonic mean (F1-score), and the mean average precision (mAP) score. These metrics are formally defined as follows:

$$\mathrm{PPV}=\frac{TP}{TP+FP}$$

$$\mathrm{Sensitivity}=\frac{TP}{TP+FN}$$

$$F1\text{-}\mathrm{score}=2\times \frac{\mathrm{PPV}\times \mathrm{Sensitivity}}{\mathrm{PPV}+\mathrm{Sensitivity}}$$

$$\mathrm{mAP}=\overset{1}{\underset{0}{\int }}\;\mathrm{PPV}(\mathrm{Sensitivity})\;d(\mathrm{Sensitivity})$$

Because polyp detection in this study constitutes a single-class object detection task, the mAP-score is equivalent to the Average Precision (AP), representing the exact area under the PPV-Sensitivity (precision-recall) curve. Thus, the mAP-score reflects the model's overall ability to correctly identify true polyps while minimizing false detections across all internal confidence thresholds. To evaluate the model's overall detection capability, all performance metrics were calculated by globally aggregating the TP, FP, and FN occurrences across all images before applying the formulas above. The reported PPV, Sensitivity, and F1-score were established using the specific internal confidence threshold that yielded the maximal F1-score (where a high F1-score indicates that both PPV and Sensitivity are high and balanced; large differences between the two metrics decrease the score). Detection metrics are reported as means with standard deviation. One-Way ANOVA compared metrics between models, followed by Tukey's HSD *post-hoc* analysis. Significance was set at a *p*-value < 0.05. Statistical analyses were performed using SciPy, version 1.8.0.

### Implementation details and reproducibility

All models, processing pipelines, and statistical analyses were implemented using Python 3.8. Computational tasks were executed on the Hábrók High-Performance Computing cluster at the University of Groningen. Specifically, the generative adversarial networks (GANs) and the YOLOv5m detection models were implemented using PyTorch 1.10. The GANs were trained utilizing two NVIDIA A100 GPUs, while the detection models were trained using a single NVIDIA V100 GPU. Image feature extraction via ViT was conducted using the PyTorch Image Models (*timm*) package (version 0.9.12), while scikit-learn (version 1.3.0) supported SVM implementation, k-means clustering, and t-SNE visualizations.

Prior to training, all images from the UMCG, PIBA, and SUN databases were resized to 512 × 512 pixels. For StyleGAN2-ADA training, pixel values were normalized to the range of [–1, 1]. Models were trained for 6 million images shown to the discriminator using a batch size of 16 and the Adam optimizer (learning rate: 0.0025; β_1_: 0; β_2_: 0.99). Detailed configurations are provided in Supplementary Material S4. For YOLOv5m detection models, pixel values were normalized to [0, 1]. These models were trained for 100 epochs with a batch size of 64 using the Stochastic Gradient Descent (SGD) optimizer (initial learning rate: 0.01; momentum: 0.937; weight decay: 0.0005) Further training specifications are available in Supplementary Material S5.

## Results

### Ablation study and model selection

To validate our feature-clustered conditioning method against standard approaches and to determine the optimal architecture for our modified StyleGAN2-ADA, we conducted an ablation study (Table 2). We systematically tested combinations of conditioning strategies and technical modifications. For the embedding strategies, we compared our proposed ViT centroid embeddings against a baseline of simple one-hot encoded labels representing the identified clusters. We tested these conditioning strategies alongside three specific technical modifications: hinge loss, classifier guidance loss, and the StyleSplit method. In this setup, configurations A–C evaluated the simpler one-hot labels paired with various combinations of our technical modifications, while the remaining configurations evaluated our ViT centroids. The optimal configuration, which utilized the ViT centroids combined with all three technical modifications (hinge loss, classifier guidance loss, and StyleSplit), achieved the lowest (best) FID score of 7.54 (configuration H). These findings suggest that the additional sub-class information provided by the feature-clustered embeddings can further enhance synthesis control and image fidelity within the fully optimized architecture.

**Table 2:** Ablation study results of various configurations.

**Config.**	**ViT centroids**	**Hinge**	**SS**	**CLF**	**FID**
A	–	–	–	–	10.7
B	–	✓	–	–	8.24
C	–	✓	–	✓	9.86
D	✓	–	–	–	10.5
E	✓	✓	–	–	8.85
F	✓	✓	✓	–	9.24
G	✓	✓	–	✓	9.35
H	**✓**	**✓**	**✓**	**✓**	**7**.**54**

Configurations A–C show the FID scores of the conditional StyleGAN2-ADA models with conditioning one-hot labels are used for the found ViT clusters. The other configurations show the results of the models utilizing the centroid embeddings as conditioning. Hinge: Hinge loss, SS, Stylesplit, CLF, Classifier Guidance Loss. The model configuration with the best obtained FID score is highlighted in bold (configuration H).

Bold values indicate the model configuration with the best (lowest) obtained FID score.

### Synthetic data generation

As established in the ablation study, the lowest FID score achieved during training was 7.54 for the modified StyleGAN2-ADA, compared to 13.68 for the original StyleGAN2-ADA. To generate synthetic datasets approximating 80% of the size of the UMCG database, a total of 22,500 images were synthesized by the original StyleGAN2-ADA, yielding 1,820 (8.1%) images selected for use, and 21,000 images were synthesized by the modified StyleGAN2-ADA, with 1,782 (8.5%) selected.

**Figure 3:**
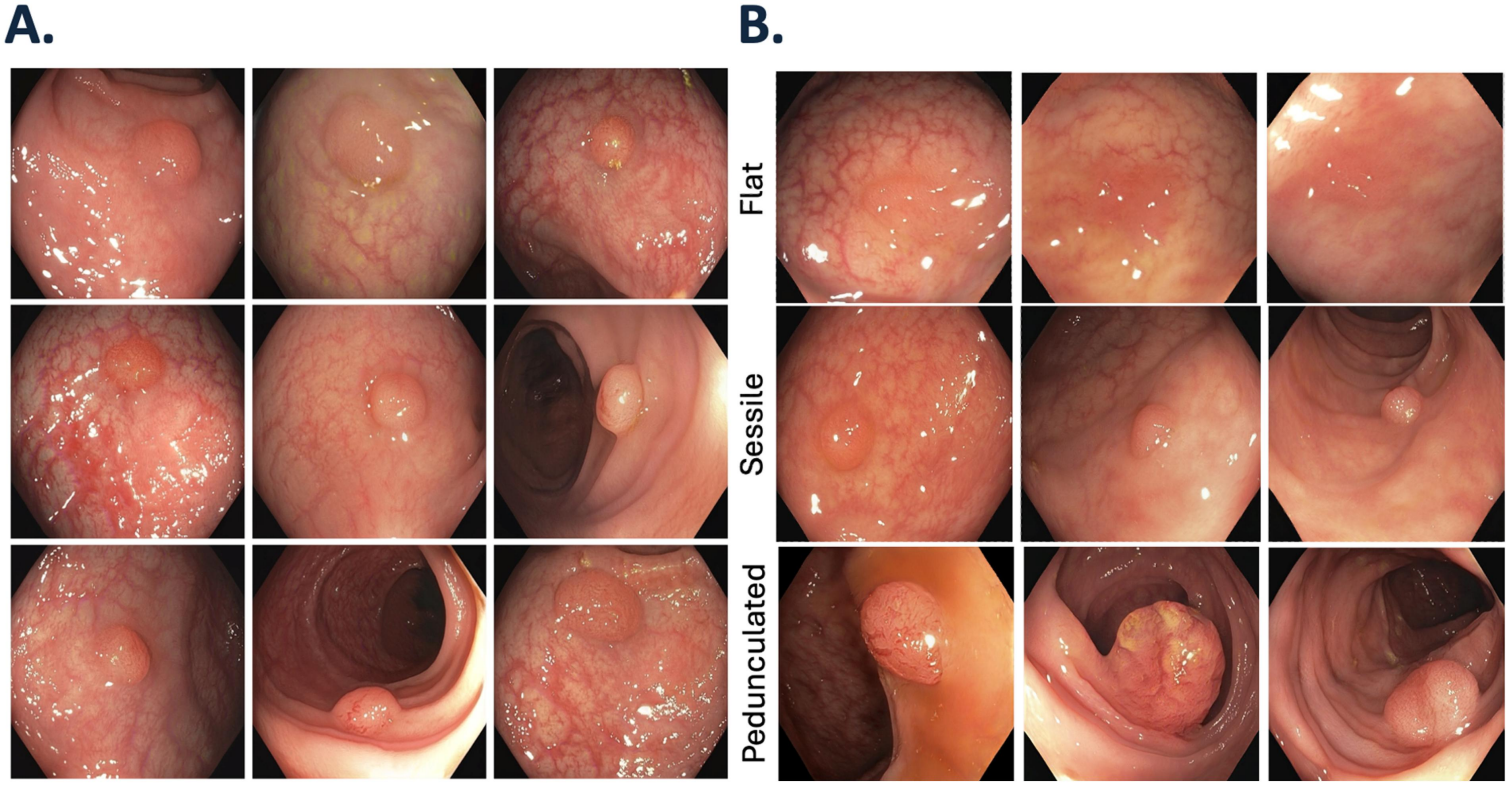
Output by the GAN models after manual selection. **(A)** Output of the unconditional StyleGAN2-ADA, showcasing overall similar polyp morphology in the synthetic data. **(B)** Output of the modified conditional StyleGAN2-ADA, conditioned on ViT-feature clusters. Three distinct examples of the flat, sessile, and pedunculated polyp class are shown.

From the synthetic dataset generated by the original StyleGAN2-ADA, 131 (7.2%) images were manually labeled as flat, 1,690 (92.9%) images as sessile, and 2 (0.1%) images as pedunculated, resulting in a class distribution comparable to that of the initial real-world dataset. Conversely, from the synthetic dataset generated by the modified StyleGAN2-ADA, 682 (38.3%) images were manually labeled as flat, 996 (55.9%) images as sessile, and 104 (5.8%) images as pedunculated, mitigating the original class imbalance. These manual labels corresponded to the labels of the intended polyp class (that the feature clusters represented for image generation) in 76.8% of the flat, 77.6% of the sessile, and 84.6% of the pedunculated cases. Figure 3 displays random samples from the selected synthetic images generated by both GAN versions.

### Detection performances

#### Detection performances under the three training conditions

**Figure 4:**
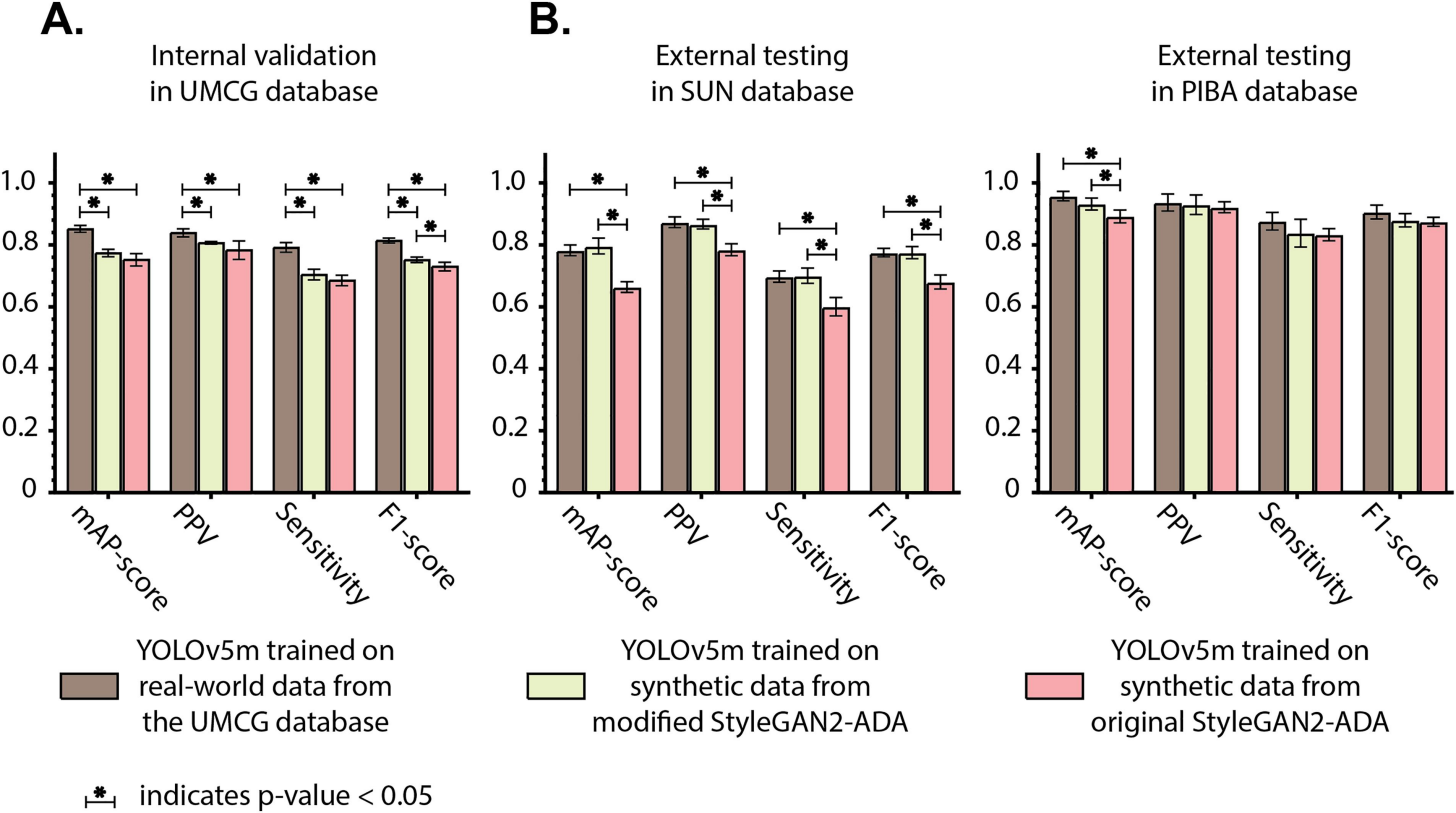
Overview of the variously trained YOLOv5m models. **(A)** Detection performances during internal validation in the UMCG database. **(B)** Detection performances during external testing in the SUN and PIBA databases.

Figure 4 displays the results of the YOLOv5m models that were trained using real-world data from the UMCG database, synthetic data from the original StyleGAN2-ADA, and synthetic data from the modified StyleGAN2-ADA. During internal validation (Figure 4A), YOLOv5m trained on real-world data outperformed both models trained on synthetic data across all metrics (*p* < 0.05). Furthermore, YOLOv5m trained on synthetic data from the modified StyleGAN2-ADA tended to surpass the model trained on synthetic data from the original StyleGAN2-ADA, achieving statistical significance for the F1-score (75.2% (±0.9) vs. 73% (±1.4), *p* = 0.019).

In both external databases (Figure 4B), YOLOv5m trained on synthetic data from the modified StyleGAN2-ADA achieved performances comparable to the model trained on real-world data (*p* > 0.05). In the SUN database, it was superior to the model trained on synthetic data from the original StyleGAN2-ADA across all metrics (PPV: 84.3% (±1.5) vs. 76.2% (±1.9), *p* < 0.001; sensitivity: 68.0% (±2.4) vs. 58.2% (±2.9), *p* < 0.001; F1-score: 0.75 (±0.02) vs. 0.66 (±0.02), *p* < 0.001; and mAP-score: 0.77 (±0.03) vs. 0.64 (±0.02), *p* < 0.001). In the PIBA database, YOLOv5m trained on synthetic data from the modified StyleGAN2-ADA was superior to the model trained on synthetic data from the original StyleGAN2-ADA regarding mAP-score (0.91 (±0.02) vs. 0.87 (±0.02), *p* = 0.012)).

#### Detection performances after synthetic augmentation

No significant improvement was observed during internal validation for augmentation of the database with synthetic images (Table 3).

**Table 3:** Baseline performance with traditional augmentation and performance after synthetic augmentation in combination with traditional augmentation of YOLOv5m models.

**Database**	**Training condition**	**mAP-score**	**PPV**	**Sensitivity**	**F1-score**
UMCG database	Authentic data	0.85 ± 0.01	83.9% ± 1.3	79.2% ± 1.6	0.81 ± 0.01
	Augmented with data from original StyleGAN2-ADA	0.86 ± 0.01	82.8% ± 2.7	79.9% ± 2.1	0.81 ± 0.02
	Augmented with data from modified StyleGAN2-ADA	0.86 ± 0.01	84.0% ± 1.5	79.6% ± 0.7	0.82 ± 0.01
SUN database	Authentic data	0.76 ± 0.02	84.9% ± 1.7	67.7% ± 1.8	0.75 ± 0.01
	Augmented with data from original StyleGAN2-ADA	0.76 ± 0.03	84.8% ± 1.6	67.8% ± 3.6	0.75 ± 0.03
	Augmented with data from modified StyleGAN2-ADA	0.79 ± 0.03	85.6% ± 1.4	70.0% ± 2.6	0.77 ± 0.02
	Augmented with data from centroids of flat polyp class	0.78 ± 0.03	84.8% ± 1.7	69.4% ± 3.1	0.76 ± 0.02
PIBA database	Authentic data	0.93 ± 0.02	91.2% ± 2.7	85.3% ± 2.8	0.88 ± 0.02
	Augmented with data from original StyleGAN2-ADA	0.94 ± 0.02	91.7% ± 1.9	88.9% ± 4.7	0.90 ± 0.03
	Augmented with data from modified StyleGAN2-ADA	0.94 ± 0.01	93.7% ± 2.0	84.5% ± 2.0	0.89 ± 0.01
	Augmented with data from centroids of pedunculated polyp class	0.94 ± 0.01	92.7% ± 1.9	87.9% ± 1.9	0.90 ± 0.01

## Discussion

This study demonstrates that synthetic datasets can serve as readily accessible alternatives to real-world databases for CADe training. For synthetic data generation, we evaluated both the original StyleGAN2-ADA and a modified version with feature-clustered conditioning vectors. Each network produced datasets suitable for training YOLOv5m, although only subsets of their total output were useful. Whereas the original GAN largely reproduced the imbalanced distribution of real-world data, the modified GAN yielded a more balanced dataset by synthesizing a wider range of polyp morphologies. Both synthetic datasets were then used as alternative training conditions to real-world data. During external validation, YOLOv5m trained on synthetic data from the modified StyleGAN2-ADA achieved performance comparable to training on real-world data and outperformed the model that was trained on synthetic data from the original StyleGAN2-ADA. Finally, we exploratively assessed whether augmenting training sets with synthetic images of underrepresented, visually hard-to-detect polyp types could further improve CADe performance. Although no statistically significant gain was observed, this concept illustrates a potential direction for future, more rigorously designed studies to investigate this application.

Over the past years, several studies have investigated GAN-generated synthetic images for training detection algorithms in colonoscopy. Shin et al. first explored a patch-based synthesis approach in 2018, integrating separate polyp lesion patches into normal mucosa backgrounds (25). This method allowed random placement of polyps but did not enable control over their morphology. In 2022, Thambawita et al. developed a non-conventional GAN architecture (SinGAN-Seg) that could generate synthetic datasets, which was particularly beneficial in settings with limited training data for polyp segmentation tasks (26). Segmentation models that were solely trained with this synthetic data achieved performance nearly equivalent to models trained on real-world data, highlighting the potential of synthetic datasets as alternative resources. Synthetic image generation for colonoscopy has also been evaluated as a data augmentation method for CADe training by Sasmal et al. (2018) and Adjei et al. (2022). While synthetic and traditional augmentation achieved comparable improvements in detection performance, combining the two did not yield consistent superiority over either method used alone (27, 28). Additionally, Yoon et al. (2022) examined whether augmenting the training database with synthetic images of sessile serrated lesions (SSLs) could improve detection performance for this rare polyp class (29). To generate synthetic data, they trained the predecessor of StyleGAN2-ADA (StyleGAN2) on images containing SSLs, which were also included in training the detection model (YOLOv3). In a separate validation set of 130 images containing SSLs, YOLOv3 trained using the synthetic augmentation method established a mAP-score of 0.93 (0.89–0.97) and YOLOv3 trained using the traditional augmentation method achieved a comparable mAP-score of 0.90 (0.85–0.95). These findings suggest that augmenting training datasets with synthetic examples of underrepresented lesions can improve detection performance. However, as in our data, conclusive evidence for superiority over traditional augmentation was not established. Lin et al. showed that a hybrid augmentation strategy, combining traditional and synthetic augmentation of real images, improved CADe performance (30). In our study, we observed a similar trend towards improved detection when adding synthetic data, although these differences did not reach statistical significance and should therefore be interpreted with caution.

This study contributes to the field by identifying the limitations of GAN architectures in colonoscopy and presenting a modified StyleGAN2-ADA capable of generating a more diverse synthetic dataset as an alternative training resource. The first limitation concerned the overall quality of the total generated output from both the original and modified GAN, necessitating manual review. In our study, only 8.1% and 8.5% of the images generated by the original and modified StyleGAN2-ADA, respectively, were retained after expert review for inclusion in the synthetic training datasets for CADe training. This requirement for manual quality control affects the apparent efficiency of large-scale GAN-based data generation to include underrepresented cases into training datasets. Nevertheless, the numbers of flat polyps in these synthetic datasets exceeded those in the real-world dataset. This renders the additional effort justifiable in contexts where the principal objective is to enhance representation of underrepresented yet clinically relevant lesion types in the training data. Therefore, we established a network that allows targeted generation of synthetic data and so, also addresses the second limitation of the original StyleGAN2-ADA. Following manual review of its output, a lack of diversity in the synthetic data was noted. This can be attributed to mode collapse: a phenomenon in which the generator becomes overly focused on a narrow set of dominant patterns, subsequently producing repetitive samples and failing to reflect the full diversity of the data distribution (31). Consequently, the synthetic dataset was strongly dominated by sessile polyps (92.9%), while flat polyps accounted for 7.2% of the generated data, reflecting the imbalanced distribution in the UMCG database that was used as input. Conversely, our modified GAN generated greater diversity across polyp morphologies, resulting in a dataset comprising 38.3% flat polyps and 55.9% sessile polyps. The improved diversity of the generated outputs was supported by a lower FID score for the modified StyleGAN2-ADA compared with the original version (7.54 vs. 13.68), indicating more realistic and diverse synthetic images. Moreover, in external testing, YOLOv5m trained on the synthetic data from the modified StyleGAN2-ADA surpassed the performance of the model trained on the synthetic data from the original StyleGAN2-ADA. Notably, it also achieved performance comparable to the performance of the model trained on real-world data, effectively bridging the performance gap between these two training resources.

This study was subject to several constraints. First, polyp detection was performed using YOLOv5, which is used for general objection detection tasks, rather than a CADe system specifically validated for colonoscopic use. This approach may have affected the absolute performance metrics in the test datasets. Second, interpretation of the internal validation results is limited by two factors: the risk of overfitting, as both GAN-based image generation and YOLOv5 training were performed solely with the UMCG database; and the risk of cross-fold data contamination, as required anonymization of UMCG images prevented patient-level separation across the validation folds. To address these issues, we performed external testing in the SUN and PIBA databases. Thus, providing a more robust performance evaluation and enabling meaningful comparison across the three training conditions. By contrast, the uneven distribution of the number of images per unique polyp in both the external databases constrained a detailed assessment of the impact of synthetic augmentation with underrepresented polyp types. Moreover, the strategy used to identify hard-to-detect lesions as potential targets for synthetic augmentation relied on subjective visual judgment rather than a reproducible selection protocol. As such, the secondary analysis should be viewed as a preliminary proof-of-concept, intended to illustrate a possible use case for targeted synthetic augmentation.

Lastly, we did not record interobserver agreement statistics for labeling polyp morphology. Consequently, we cannot quantify potential disagreement between readers, and some degree of label noise may have been introduced into the training data, particularly for borderline cases such as flat vs. sessile lesions. Such bias may also have been introduced in the GAN-generated output due to the need of manual selection to compile synthetic datasets. Constraints introduced by manual selection could be reduced by making synthetic image generation more efficient, for example by adopting architectures with multiple independent discriminators, such as StyleGAN-XL (32). Alternatively, while diffusion models have recently demonstrated good fidelity and diversity compared to GANs in benchmark experiments outside medical imaging, they typically require large datasets and substantially higher computational resources for training and inference (33, 34). Additionally, integrating advanced conditional fusion mechanisms may enable automatic bounding-box annotation in synthetic images, thereby further enhancing the utility of GAN-generated datasets for training purposes. Given the rapid evolution in generative modelling, our work should be regarded as an initial step. Thus, we anticipate that future improvements in generative architectures and auxiliary quality-control algorithms will progressively reduce dependence on manual selection and improve the practical scalability of synthetic data generation. Notably, our proposed feature-clustered conditioning mechanism is essentially architecture-agnostic which could be integrated into future diffusion models to guide the targeted synthesis of underrepresented lesions.

## Conclusion

In conclusion, this study highlights both the challenges and opportunities of using GANs to generate synthetic colonoscopy images containing polyps. Advances in GAN architectures are necessary to eliminate the labor-intensive manual review required for assembling high-quality image datasets at scale. Nevertheless, we successfully modified StyleGAN2-ADA by incorporating feature-clustered conditioning vectors to generate a high-quality, diverse synthetic dataset that can serve as an alternative to real-world data for CADe development. Moreover, our approach has the potential to enable the targeted generation of flat polyps, which are typically underrepresented in current CADe training datasets and therefore prone to be missed. Overall, our findings suggest that the proposed method may help to construct more balanced datasets and expand available training and benchmarking resources for CADe algorithms, particularly in settings where flat polyps are underrepresented in real-world data.

## Data Availability

The code used for training the generative adversariPublicly available datasets analyzed in this study can be found at their respective public repositories: the SUN database (http://amed8k.sundatabase.org/) and the PIBA database (https://www.iisgaliciasur.es/apoyo-a-la-investigacion/biobanco/colecciones/cohorte-de-imagenes-de-polipos-colorrectales-pibadb/). The raw clinical colonoscopy images from the UMCG database are not readily available due to local privacy and institutional data protection regulations. Requests to access these data should be directed to the corresponding author, W. B. Nagengast, w.b.nagengast@umcg.nl.
