# Supplementary material for: Generative adversarial networks for balancing and expanding data resources for computer-aided detection in colonoscopy
Source: Front Med Technol. 2026 Jul 9;8:1824811. doi: 10.3389/fmedt.2026.1824811 (PMC13391578; doi:10.3389/fmedt.2026.1824811)
Supplement: Supplementary file 1 [file Supplementaryfile1.docx]

**SUPPLEMENTARY MATERIAL 1: Initial non-destructive augmentation of UMCG data for GAN training**

Because the flat and pedunculated polyp classes were underrepresented in the UMCG data, they were augmented using a non-destructive approach for training of the GANs. For each of the 99 authentic flat images, 10 new images were generated, resulting in a total of 1,089 flat images. Similarly, for each of the 19 pedunculated images, 20 new images were created, resulting in a total of 399 pedunculated images. The augmentation pipeline consisted of the following transforms:

- Horizontal and vertical flips.
- Grid distortion.
- Rotation in the 360-degree range.
- Series of cropping and resizing operations.
- Elastic transform.

After the augmentation operations for each image, the black triangles of the colonoscopic view were added in each of the four corners, so the augmented images closely resemble the overall look of the original images. An example of an original flat image and its 10 initially augmented images is shown in Supplementary Figure 1.

**Supplementary Figure 1.** Example of the initial augmentation method.


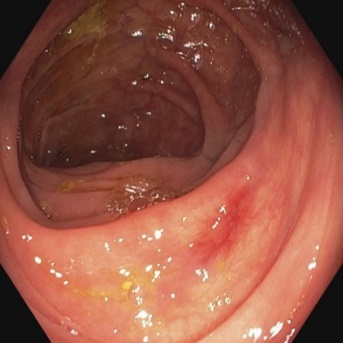

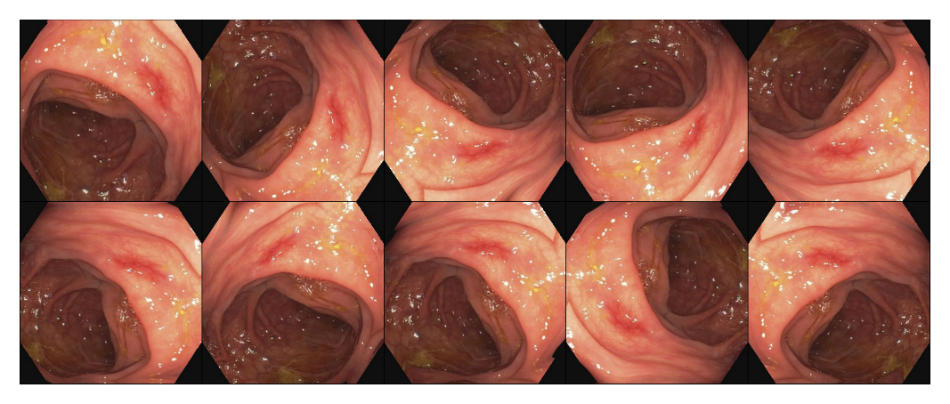


*The original image is shown on the left, while 10 augmented images are shown on the right.*

Additionally, images from the no polyp and sessile classes are augmented by applying a horizontal flip. This effectively doubles the number of images for these classes. After these initial augmentations, the dataset consists of 8,614 images in total, used for training the GANs.

**SUPPLEMENTARY MATERIAL 2: Image feature extraction for modifying StyleGAN2-ADA**

StyleGAN2(-ADA) has previously (by the authors of StyleGAN2(-ADA)) been made class-conditional by embedding one-hot encoded class labels into a 512-dimensional space. However, due to the homogeneous nature of colonoscopy images and the inability of one-hot labels to account for intra-class variations, this approach was deemed insufficient for effective conditioning the generation process. To address this limitation, we employed richer sub-class embeddings beyond simple class labels, by extracting image features using pre-trained models.

We evaluated how informative extracted pre-trained features of the UMCG colonoscopy images are, by observing the separability of the four main classes in the feature space of the last layer of two pre-trained models: ResNet50^1^ and ViT^2^. Both models were pre-trained on ImageNet-1k. The resulting feature embeddings have dimensions of 2048 (ResNet50) and 768 (ViT). SVMs with 10-fold cross-validation were trained on these features and t-SNE^3^ was performed to visualize the features in 2D.

**Supplementary** **Table 1.** Results of 10-fold cross-validation using an SVM on the image features obtained using two different pre-trained models.

| **Model** | **Accuracy** | **F1** |
| --- | --- | --- |
| ViT | 0.878 ± 0.059 | 0.875 ± 0.084 |
| ResNet50 | 0.826 ± 0.062 | 0.809 ± 0.098 |

Supplementary Table 1 presents the results of the 10-fold cross-validation of the SVM classifier applied on the ViT and ResNet50 features of the images of the UMCG dataset. Performance evaluation of the cross-validation is based on the mean accuracy and F1-score computed across all 10 runs. To test statistical differences, paired t-tests (with a significance level of P ≤ 0.05) were performed. Empirical observations revealed that employing a Radial Basis Function (RBF) kernel with a regularization parameter C set at 10 yielded the best SVM results in general. The results show that SVMs trained with ViT features and ResNet50 features both achieve accuracies and F1-scores above 80%. The results also show that the ViT features achieved significantly higher accuracy and F1-score (P < 0.001 for both metrics) compared to the ResNet50. Additionally, ViT features have a lower dimensionality (768 vs. 2048). These findings suggest that ViT features are more informative and suitable as conditional embeddings for a conditional GAN model compared to the ResNet50 features.

**Supplementary** **Figure 2.** 2D t-SNE plot of the extracted ViT features of the colonoscopy images. Different colors highlight the four different main classes*.*


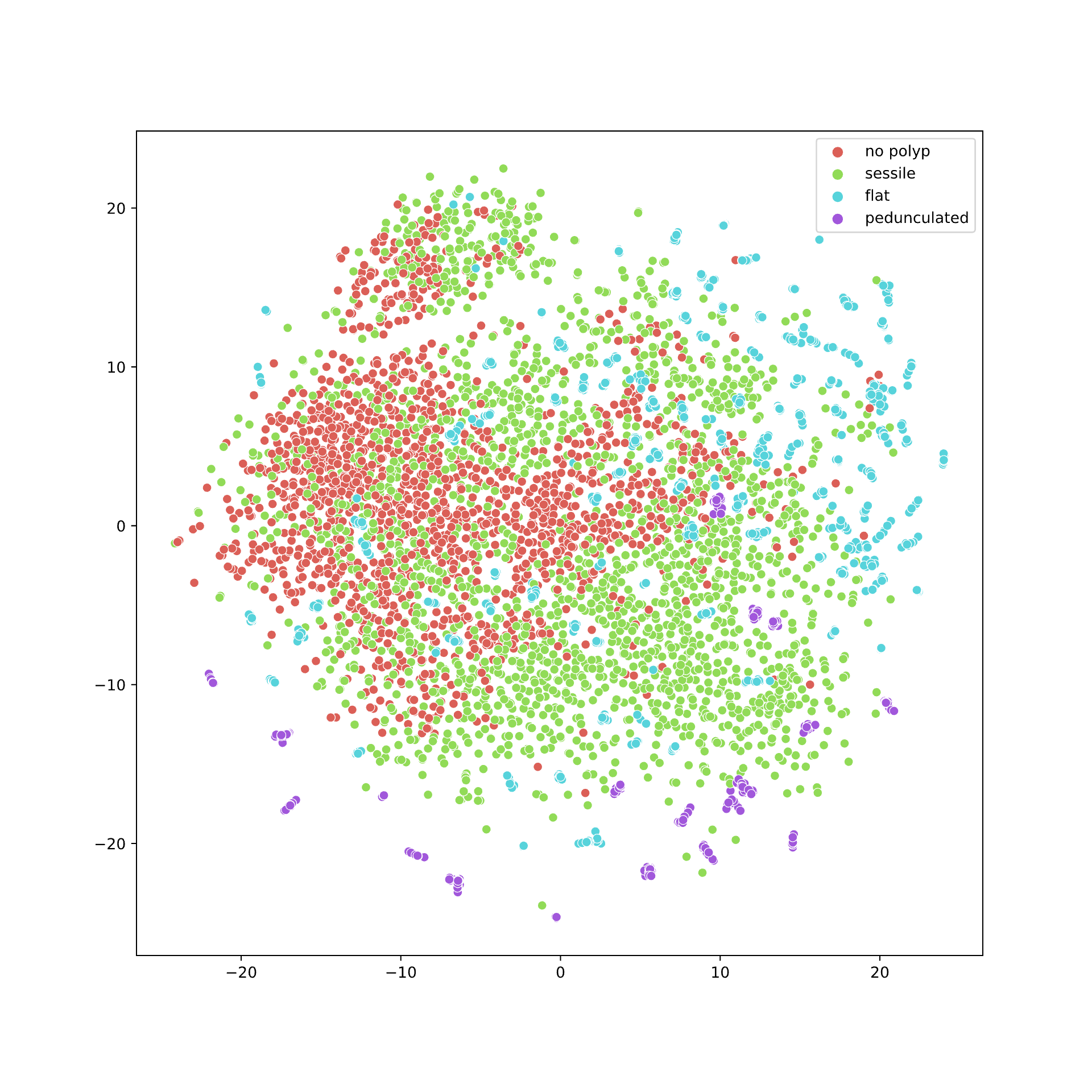


Supplementary Figure 2 shows the t-SNE plot of ViT features extracted from the UMCG dataset, where the different classes are mostly separable. Based on this, the ViT features were be used to obtain the conditional embeddings for the GAN.

After feature extraction, K-means clustering was applied to the image features for deriving *k* clusters of each main polyp class. Each cluster is represented by a cluster that encompasses distinct feature variations within the class and serve as conditioning vectors to control the subclasses of the polyp classes. Ultimately, we adapted the StyleGAN2-ADA model to use the 768-dimensional ViT centroids, which were mapped to a 512-dimensional conditioning vector, using a fully connected layer.

**SUPPLEMENTARY MATERIAL 3: Technical additions conditional StyleGAN2-ADA**

Additional modifications were implemented to optimize generation outcomes. First of all, the model switched from logistic loss to hinge loss.^4^

Secondly, deduced from StyleGAN-XL^5^, a classifier guidance loss term was introduced to better guide the generator. In Supplementary Material B it has been shown that the feature extractor of the ViT model, in combination with an SVM classifier is very capable of separating the four different main classes in the dataset. Because of this, there is chosen for this feature extractor and classifier. The SVM model for this classifier guidance loss is trained with the ViT features obtained on all image in the UMCG dataset after initial augmentation. For the SVM the regularization parameter C is set to 10 and the RBF kernel is used. To compute the classifier guidance loss during training, features of generated images (resized to 224x224) are extracted using ViT, and cross-entropy loss is computed between the predicted (obtained using the SVM) and ground truth (main class the ViT centroid belongs to) labels as shown in the equation below. Here, the ground truth classes are *c_i_* and the generated images are *â_i_*. The resulting loss is scaled by a constant hyperparameter $\lambda_{1}$ and added as an additional term to the generator loss. It is important to note that the weights of the ViT model are fixed. Mathematically $L_{clf}$ is described as:

$$L_{clf}=-\sum_{i=0}^{C} c_{i}log\left( CLF\left( \hat{a_{i}} \right) \right)$$

Thus, the complete generator and discriminator losses can be described as follows:

$$L_{D}=-E_{\left( x,c \right)\sim P_{data}\left( x \right)}\left[ min\left( 0,-1+D\left( x,c \right) \right) \right]-E_{z\sim P_{z},c\sim P_{data}}\left[ min\left( 0,-1-D\left( G\left( z,c \right),c \right) \right) \right]+\gamma R_{1}$$

$$L_{G}=-E_{z\sim P_{z},c\sim P_{data}}D\left( G\left( z,c \right),c \right)+\lambda_{1}L_{clf}$$

With $c$ as a ViT centroid for conditioning,$R_{1}$ as the gradient penalty^6^ which is scaled by hyperparameter $\gamma$ and $L_{clf}$ as the classifier guidance loss, scaled by hyperparameter $\lambda_{1}$. Here, $P_{\mathrm{data}}$ denotes the true data distribution from which real samples $x$ and their corresponding conditions $c$ are drawn. The noise vector $z$ is is sampled from $P_{z}$ and Generator $G\left( z,c \right)$ generates a synthetic sampled conditioned on $c$. Discriminator $D\left( x,c \right)$ score samples for authenticity.^7^

The last addition is the adoption of the *Stylesplit* method, introduced in StyleGAN-T^8^, for better fusion of conditional embeddings instead of a single linear layer.

**SUPPLEMENTARY MATERIAL 4: GAN training hyperparameters**

Supplementary Table 2 shows the hyperparameters used for training the StyleGAN2-ADA models. The classifier guidance loss weight is only applicable to the modified conditional StyleGAN2-ADA model. Training continued for 6000k images shown to the discriminator, with FID^9^ evaluations every 500k images. The checkpoint with the lowest FID score was used for sampling for the two models.

**Supplementary Table 2.** Hyperparameters used to train the original and modified GAN model.

| **Hyperparameter** | **Value** |
| --- | --- |
| Mapping Net Depth | 2 |
| D Backbone | ResNet |
| G Backbone | Skip |
| Learning Rate D | 0.0025 |
| Learning Rate G | 0.0025 |
| Optimizer | Adam: β1 = 0, β2 = 0.99 |
| Style Mixing | 0.9 |
| Path Length | 2 |
| Z Dim | 512 |
| W Dim | 512 |
| Loss function | Non-saturating logistics loss |
| R1-regularization | 3.2768 |
| Batch Size | 16 |
| ADA | Pixel blitting, geometric, color |
| Training length | 6000 kimg |
| λ_1_ (Classifier guidance loss weight) | 4 |

**SUPPLEMENTARY MATERIAL 5: YOLOv5 training hyperparameters**

Supplementary Table 3 shows the hyperparameters used for training the CADe YOLOv5m model for all experiments.

**Supplementary Table 3.**

| **Hyperparameter** | **Value** |
| --- | --- |
| Learning Rate Initial | 0.01 |
| Learning Rate Final | 0.01 |
| Optimizer | SGD : momentum=0.937 |
| Weight Decay | 0.0005 |
| Warmup Epochs | 3 |
| Warmup Momentum | 0.8 |
| Warmup Bias Learning Rate | 0.1 |
| Box Loss Gain | 0.05 |
| Cls Loss Gain | 0.5 |
| Cls BCE Loss Positive Weight | 1.0 |
| Objectness Loss Gain | 1.0 |
| Objectness BCE Loss Positive Weight | 1.0 |
| IoU Training Threshold | 0.20 |
| Anchor-Multiple Treshold | 4.0 |
| Focal Loss Gamma | 0.0 |
| Batch Size | 64 |
| Number of Epochs | 100 |
| HSV Hue | 0.015 |
| HSV Saturation | 0.7 |
| HSV Value | 0.4 |
| Rotation | 0.0 |
| Translation | 0.1 |
| Scale | 0.5 |
| Shear | 0 |
| Perspective | 0 |
| Horizontal Flip | 0.5 |
| Vertical Flip | 0.5 |
| Mosaic | 1 |
| Mixup | 0 |
| Copy Paste | 0 |

YOLOv5 hyperparameters for all training experiments. Above the bold horizontal line general hyperparameters are shown, below are the hyperparameters for traditional data augmentation.

**SUPPLEMENTARY REFERENCES**

1. He, K., Zhang, X., Ren, S. & Sun, J. Deep Residual Learning for Image Recognition. in *2016 IEEE Conference on Computer Vision and Pattern Recognition (CVPR)* 770–778 (2016). doi:10.1109/CVPR.2016.90.

2. Dosovitskiy, A. *et al.* An Image is Worth 16x16 Words: Transformers for Image Recognition at Scale. Preprint at http://arxiv.org/abs/2010.11929 (2021).

3. Maaten, L. van der & Hinton, G. Visualizing Data using t-SNE. *J. Mach. Learn. Res.* **9**, 2579–2605 (2008).

4. Lim, J. H. & Ye, J. C. Geometric GAN. Preprint at http://arxiv.org/abs/1705.02894 (2017).

5. StyleGAN-XL: Scaling StyleGAN to Large Diverse Datasets | ACM SIGGRAPH 2022 Conference Proceedings. https://dl.acm.org/doi/10.1145/3528233.3530738.

6. Mescheder, L., Geiger, A. & Nowozin, S. Which Training Methods for GANs do actually Converge? in *Proceedings of the 35th International Conference on Machine Learning* 3481–3490 (PMLR, 2018).

7. Mirza, M. & Osindero, S. Conditional Generative Adversarial Nets. Preprint at http://arxiv.org/abs/1411.1784 (2014).

8. Sauer, A., Karras, T., Laine, S., Geiger, A. & Aila, T. StyleGAN-T: unlocking the power of GANs for fast large-scale text-to-image synthesis. in *Proceedings of the 40th International Conference on Machine Learning* vol. 202 30105–30118 (JMLR.org, Honolulu, Hawaii, USA, 2023).

9. Heusel, M., Ramsauer, H., Unterthiner, T., Nessler, B. & Hochreiter, S. GANs trained by a two time-scale update rule converge to a local nash equilibrium. in *Proceedings of the 31st International Conference on Neural Information Processing Systems* 6629–6640 (Curran Associates Inc., Red Hook, NY, USA, 2017).
